# Detection of Hepatitis E Virus in Game Meat (Wild Boar) Supply Chain in Umbria Region, Central Italy

**DOI:** 10.3390/foods13162504

**Published:** 2024-08-09

**Authors:** Monica Borghi, Elisa Pierboni, Sara Primavilla, Eleonora Scoccia, Claudio Costantini, Elisabetta Suffredini, Alessandro Graziani, Piero Macellari, Salvatore Macrì, Silvana Farneti, Andrea Valiani

**Affiliations:** 1 Istituto Zooprofilattico Sperimentale dell’Umbria e delle Marche “T. Rosati”, 06126 Perugia, Italy; e.pierboni@izsum.it (E.P.); s.primavilla@izsum.it (S.P.); e.scoccia@izsum.it (E.S.); s.farneti@izsum.it (S.F.); a.valiani@izsum.it (A.V.); 2 Department of Medicine and Surgery, Pathology Division, University of Perugia, L. Severi Square, 06129 Perugia, Italy; claudio.costantini@unipg.it; 3 Department of Food Safety, Nutrition and Veterinary Public Health, Istituto Superiore di Sanità, Viale Regina Elena 299, 00161 Rome, Italy; elisabetta.suffredini@iss.it; 4 Department of Medicine and Surgery, Microbiology and Clinical Microbiology Division, University of Perugia, L. Severi Square, 06129 Perugia, Italy; alessandro.graziani@dottorandi.unipg.it; 5 Regione Umbria, Direzione Salute e Welfare—Servizio Prevenzione, Sanità Veterinaria e Sicurezza Alimentare, 06124 Perugia, Italy; pmacellari@regione.umbria.it (P.M.); smacri@regione.umbria.it (S.M.)

**Keywords:** food safety, foodborne transmission, HEV, hunted wild boar (WB) meat

## Abstract

Consumption of raw or undercooked wild boar (WB) meat is considered an important risk factor for hepatitis E virus (HEV) infection in humans. The possibility of HEV contamination during the slaughtering practices may pose an additional risk. Based on these assumptions, we evaluated HEV contamination of WB meat hunted in Umbria (central Italy) during the 2022–2023 hunting season by real-time RT-PCR. Herein, we show that 10.8% of livers from slaughtered WB were positive for HEV RNA, thus providing an estimate of HEV infection in WB in the Umbria region. Then, by evaluating paired liver–muscle samples from both HEV-positive and HEV-negative animals, we found evidence of muscle HEV contamination in 33% and 14% of cases, respectively. This is the first report on the detection of HEV in WB meat in Umbria, an Italian region with diffuse WB hunting and consumption. The evidence of contamination provided by our study underscores the importance of adopting good hygienic practices in the processing stages of hunted WB carcasses to significantly reduce meat contamination and the risk posed for the final consumer.

## 1. Introduction

Hepatitis E virus (HEV) has a global distribution, and it is the most common cause of acute self-limiting hepatitis worldwide. The clinical consequences differ considerably, varying from asymptomatic infection in the vast majority of patients to fulminant hepatitis, which is associated with a high mortality rate, particularly in pregnant women in the second or third trimester [1,2,3].

Due to nonspecific symptoms and self-limited disease, hepatitis E is frequently not diagnosed [4,5]. Despite these limits, in 2023, hepatitis E was the third most frequent cause of viral hepatitis in Italy (https://www.epicentro.iss.it/epatite/dati-seieva#e, accessed on 17 April 2024).

HEV has eight genotypes (G1–G8), four of which (G1 to G4) cause infections in humans [6]. In Europe, hepatitis E is believed to be typically a zoonotic disease because it is mainly associated with HEV genotype 3 (HEV-3) and sporadically associated with genotype 4 (HEV-4), which are able to infect both humans and animals (mainly pigs and wild boar—WB) [7,8,9,10]. Zoonotic transmission occurs by direct contact with infected animals and consumption of HEV-contaminated food products [11].

In Italy, consumption of raw or undercooked WB meat, sausages, and WB liver products is considered a major risk factor for HEV infection in humans [12,13,14,15]. These products are widely consumed in central Italy, especially in some regions like Umbria, where a high prevalence and wide genetic variety of HEV-3 has been recently reported in WB populations [16]. These results were obtained through an active surveillance plan established by the health authorities of the region during the hunting season from October 2021 to January 2022. The high prevalence of HEV in WBs (43%, testing liver tissue) and the wide heterogeneity of subtypes detected in this previous study provided evidence that HEV may pose a significant risk for foodborne disease outbreaks in central Italy [16]. In the same study, the presence of HEV RNA in low concentrations (close or below the quantification limit) was also detected in seven out of nine muscle samples deriving from animals that had tested positive in the liver, though a prevalence of HEV in WB meat could not be assessed due to the lack of a specifically designed sampling plan. Considering the risk of HEV infection for the local population, the health authorities of the Umbria region established a new surveillance plan during the hunting season from October 2022 to January 2023 to gather information on the health status of hunted WB meat.

As in humans, the target organ for HEV infection in WB is the liver. However, the consumption of meat and muscle preparations could also be a risk factor for HEV infection in humans. Although the tropism of the virus for tissues other than the liver has not been demonstrated yet, it has been shown that HEV RNA can be detected in muscle samples from animals whose liver had tested positive for the presence of the viral genome [16,17,18]. Further to this, meat contamination occurs by cross-contamination during food production due to unsuitable working conditions.

To fill the knowledge gap on HEV prevalence in foods and to improve food safety [19], we aimed to collect data about the contamination of meat cuts intended for raw consumption (e.g., dry sausage or salami containing liver). Herein, we first analyzed WB livers to assess the prevalence of HEV-positive animals in Umbria during the 2022–2023 hunting season. Next, we analyzed the muscles derived from all animals whose livers tested positive for the presence of the HEV RNA, as well as a selection of muscle tissues derived from animals testing negative in the analysis of livers. The aim of this work was to search for the presence of HEV in muscle tissues to understand whether the possible presence of the virus outside the liver is exclusively attributable to the viremic state of the animal at the time of slaughter rather than depending on the cross-contamination of meat caused by incorrect slaughtering practices.

The definition of critical points in the WB meat supply chain is indeed crucial to implementing quality management systems and reducing risks to consumers.

## 2. Materials and Methods

### 2.1. Experimental Design and Sampling

**Figure 1:**
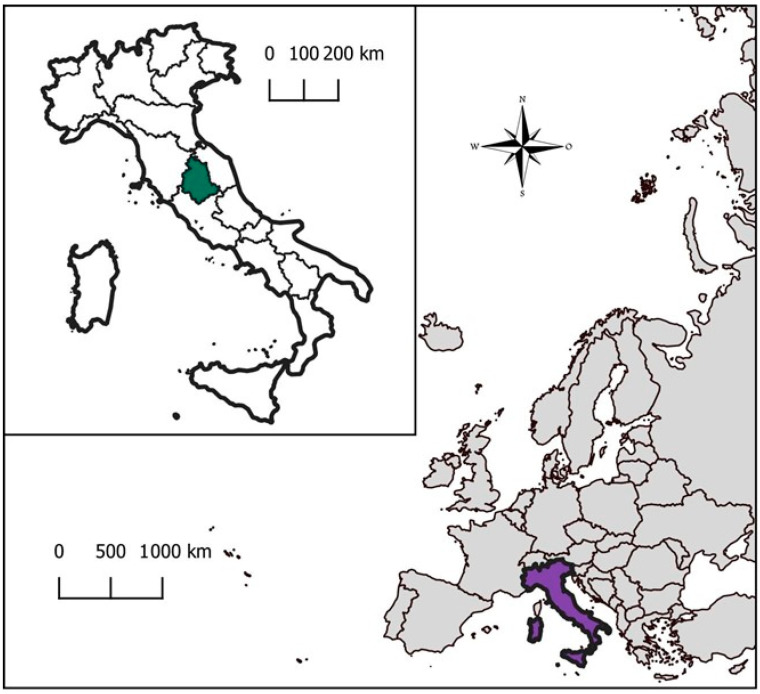
Geographical localization of Umbria region. Italy is highlighted in violet; Umbria is highlighted in green.

Collection of liver and muscle samples from wild boar (*Sus scrofa*) was performed by hunters and veterinarians actively engaged in the 2022–2023 hunting season in Umbria, central Italy (Figure 1).

To investigate the risk of HEV related to WB meat consumption in Umbria, we first quantified the prevalence of HEV-positive animals by analyzing their livers. The number of samples to be analyzed was determined by taking into account the following benchmarks: reference population (the number of wild boars slaughtered during the 2021–2022 hunting season, which was found to be 20,633); the expected prevalence of HEV infection in WB in the study area (44% as previously demonstrated [16]). This resulted in a minimum sample size of 100 livers (95% confidence level, 6% precision).

The number of WBs to be analyzed was distributed over the entire regional territory on the basis of the Umbrian hunting territorial areas (ATCs). The number of animals to be tested per ATC was calculated by taking into account the percentage of positivity estimated during the 2021–2022 hunting season [16].

Once we defined the prevalence, we also performed WB meat analyses in order to assess the presence of HEV in the muscles of animals with HEV genome-positive livers and in the muscles derived from animals with HEV-genome-negative livers.

Therefore, at the sampling stage, from each animal, 150 g of liver and a single block of muscle (preferably thigh) measuring at least 8 cm × 8 cm × 8 cm were taken simultaneously and kept separate.

To simplify the sampling procedures, a sample distribution scheme among hunting teams and a worksheet with instructions for the correct sampling were provided to all hunters.

The samples were transported to the laboratory at a controlled temperature (+4 °C) and either processed immediately or stored at −20 °C until analysis was performed.

For each sample delivered to the laboratory, a special form with the animal’s biographical information was filled out by the hunters and veterinarians.

### 2.2. Liver Samples Preparation

Liver samples were processed as previously described [20]. Briefly, 50 g of each liver sample was homogenized using a mechanical disruptor osterizer. Then, 5 g of chopped tissue was added to a 15 mL falcon tube containing 10 μL of process control virus (Mengovirus, strain MC_0_, ~10^5^ TDCI_50_/mL, kindly provided by Istituto Superiore di Sanità, National Reference Laboratory for Foodborne Viruses, Rome, Italy). Each sample was treated with 7 mL of peqGOLD TriFast™ (VWR Chemicals, Radnor, PA, USA) and vortexed at high speed for 2 min to completely homogenize the tissue. Samples were then centrifuged at 8000× *g* for 20 min at 4 °C, and the recovered supernatant was mixed with chloroform (0.2% *v*/*v*), vortexed, and incubated at room temperature for 15 min. Finally, the samples were centrifuged at 8000× *g* for 20 min at 4 °C, and the aqueous solution was recovered and measured.

A total of 1 mL of the virus preparations of each sample was used for the nucleic acid extraction by the NucliSENS^®^ easyMAG system (bioMérieux, Marcy l’Etoile, France) according to the manufacturer’s instructions. RNA was eluted in 100 μL and stored at −80 °C. A negative extraction control (molecular grade water) was added to each sample extraction batch.

### 2.3. Muscle Samples Preparation

We analyzed all muscle samples derived from the animals whose livers resulted as positive for the HEV genome. In order to detect any cross-contamination that may have occurred during animal slaughter, muscles from animals with HEV genome-negative livers were also examined. For statistical purposes, we selected a 3:1 ratio between animals with HEV-negative and HEV-positive livers, respectively.

**Figure 2:**
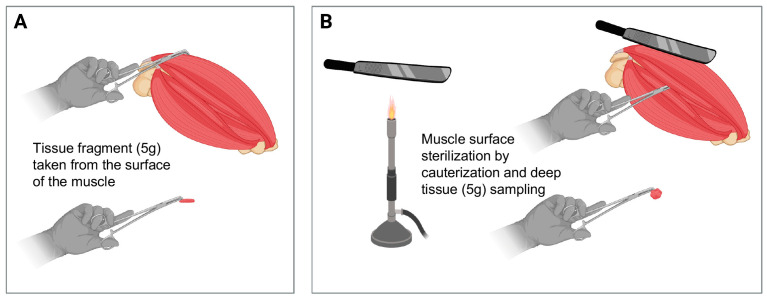
Workflow of muscle tissue sampling. The sampling was divided into two phases: (**A**) First, 5 g of tissue from the surface of the muscle block was taken. (**B**) Thereafter, the surface of the muscle was sterilized by cauterization with a hot metal spatula, and 5 g of deep tissue was also taken. The figure was created with BioRender.com.

For each selected muscle, we took 5 g of tissue from the surface of the meat block. Then, after sterilizing the muscle surface by cauterization, we also sampled 5 g of deep tissue (Figure 2).

Samples taken from the same muscle block were processed separately following the same procedure used for livers and described above (Section 2.2). To minimize the risk of cross-contamination during analysis, muscles derived from liver-negative animals (36/48) were analyzed on different days and sessions than muscles derived from liver-positive animals (12/48). Negative extraction controls (molecular grade water) were included in each sample extraction batch.

### 2.4. HEV Analysis by Real-Time RT-PCR

In order to detect the HEV genome in the samples, a qualitative real-time RT-PCR assay for the specific identification of the ORF3 region of HEV was carried out. Specifically, we used the RNA UltraSense™ One-Step qRT-PCR System (Thermo Fisher Scientific Inc., Waltham, MA, USA), as previously described [21], and the QuantStudio7 flex real-time PCR system (Thermo Fisher Scientific Inc., Waltham, MA, USA).

Each reaction was prepared using a total volume of 25 μL, containing 5 μL of the sample nucleic acids, 1.25 μL of enzyme mix, reverse primer (900 nM), forward primer (500 nM), and probe (250 nM). The RT-PCR primers and probe sequences (5′-3′) are reported in Table 1.

Each sample was tested in duplicate. A criteria-based evaluation of the presence or absence of the amplification curves of the specific amplified products was used to interpret the results: presence of amplification curve = positive sample; absence of amplification curve = negative sample.

In addition, to monitor the contamination events, a negative PCR control, a negative environmental control, and a negative extraction control were included in each analytical session. The amplification conditions were as follows: reverse transcription for 60 min at 50 °C, inactivation for 5 min at 95 °C and 45 cycles of 15 s at 95 °C, 1 min at 60 °C, and 1 min at 65 °C. Virus extraction efficiency was assessed according to Costafreda et al. [24] by comparing the threshold cycle (Cq) value of the Mengovirus RNA obtained in spiked samples with the Cq value of the viral stock used for spiking. The extraction efficiency was considered acceptable for values ≥ 1%. Moreover, a control for RT-PCR inhibition was also performed using an in vitro synthesized HEV external control RNA (EC-RNA), kindly provided by the Istituto Superiore di Sanità, National Reference Laboratory for Foodborne Viruses, Rome, Italy. The Cq value obtained from the external control RNA-added samples (Sample + EC-RNA) was compared with the Cq value obtained from the analysis of the EC-RNA alone. Samples with ΔCq < 2 were considered acceptable for inhibitors’ presence.

### 2.5. Statistical Analysis

The prevalence for HEV and the related Wilson confidence interval (95%) were calculated. A descriptive statistical analysis of the samples was carried out for the variables associated with the tested livers, such as the sex and age of the animal, the origin (municipality and relevant ATC), and the detection of HEV. Furthermore, any association was evaluated using the Pearson χ^2^ test, considering a *p*-value < 0.05 significant, and, in the case of associations, the odds ratio (OR) was calculated with the confidence interval (CI 95%). All analyses were performed using Stata statistical software (Stata statistical software, version 16, College Station, TX, USA, StataCorp, LLC), while GIS software (QGIS version 3.16-A Coruña) was used for the georeferentiation of the samples.

## 3. Results

### 3.1. Data Analysis of WB Liver Samples

**Figure 3:**
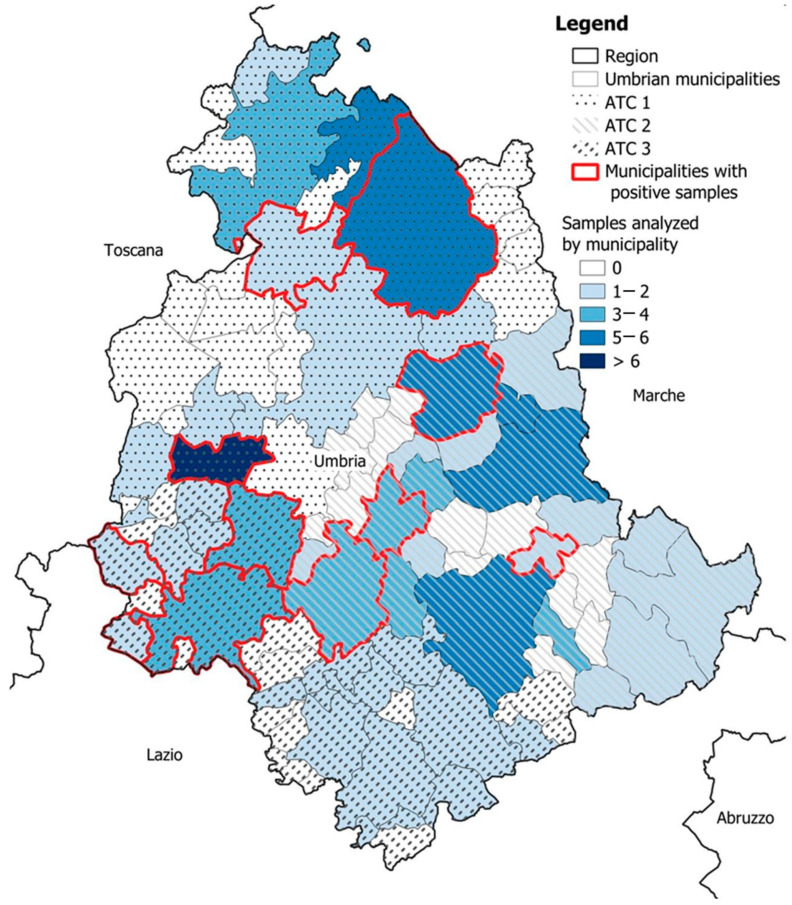
Geographical distribution of the municipalities involved in sampling with an indication of the number of samples tested for each one. Municipalities in which HEV-positive liver samples were detected are highlighted (red line).

Exceeding the number of samples intended for sampling, 111 WB liver samples were received by the laboratory between October 2022 and January 2023. All livers received were analyzed, and 12 of them resulted positive for HEV, leading to a 10.8% (IC95%: 6.3–17.9%) prevalence of hepatitis E in WB in Umbria in the hunting season 2022–2023. The distribution by municipality and the related outcome for HEV is shown in Figure 3.

The highest percentage of positive samples (Figure 3 and Table 2) was found in ATC 3 (southwest of Umbria region), with 16% of livers testing positive (4/25) compared to 9% (3/33) in both ATC1 (northern part of the region) and ATC2 (southeast) (5/53). However, the detected differences, analyzed according to the Pearson χ^2^ test, were found to be non-significant (*p*-value = 0.6365).

Based on the biographical information provided by hunters and veterinarians, most of the samples analyzed came, by chance, from male animals (64% = 71/111). The highest number of positives was found in females (13% = 5/40 compared to 10% in males); however, no statistically significant differences emerged between the two sexes (*p* = 0.6671).

When WBs were stratified by weight (information available for 105/111 of the animals), animals weighing more or less than 50 kg showed an HEV detection rate of 10% (7/71) and 12% (4/34), respectively, with no statistically significant difference (*p* = 0.7654).

Animals were also stratified according to the different age groups. Age was estimated using tooth eruption according to Matschke [25], and the animals were divided into four groups: group A (0–3 months), group B (4–12 months), group C (13–22 months), and group D (>23 months). Most of the animals belonged to age group D (66.7%:74/111), followed by 21.6% (24/111) of age group C and 9.9% (11/111) of age group B. Only 1.8% (2/111) of the animals belonged to age group A. With 25% (6/24) of positive animals, age group C was the one with the highest positivity rate for HEV. The results of the analysis of HEV in relation to age are presented in Table 3.

Finally, we tested the association between age and the presence of HEV in the livers of WB by comparing group D, with the lowest positivity, and either group C (*p* = 0.0139) or group B (*p* = 0.7779). The comparison could not be performed with group A since no positivity was detected. Our analysis showed that group C was almost 5 times more likely to have HEV-positive livers than group D (OR = 4.6; IC95% = 1.02–21.03).

### 3.2. Data Analysis of WB Muscle Samples

A total of 48 muscle samples were analyzed; specifically, 12 and 36 samples were taken from animals with HEV-positive and HEV-negative livers. Muscle samples were mostly taken from the thigh area (*n* = 42). To a lesser extent, the shoulder (*n* = 3) and loin (*n* = 2) were also sampled. For one sample, the body district was unknown.

**Figure 4:**
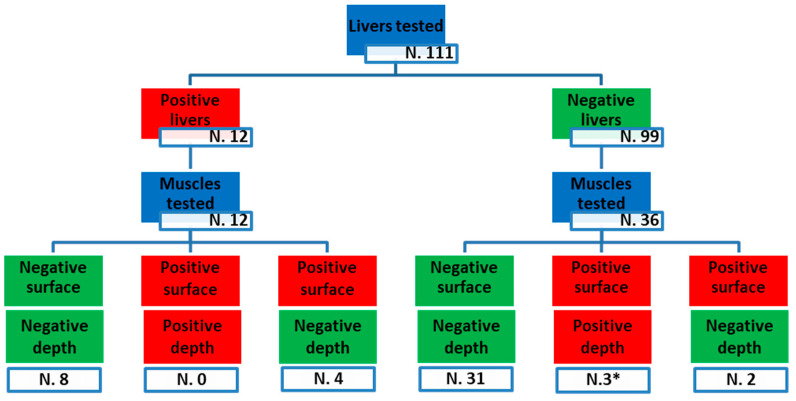
Sample flow-chart. Tissues tested are highlighted in blue; Positive samples are highlighted in red; negative samples are highlighted in green. For muscle testing, two positive results were obtained on loin tissue (marked with asterisk), and the other seven positive results were obtained on thigh tissue.

Figure 4 summarizes the flow of samples analyzed for all matrices considered: liver, tissue taken from the surface of the meat block, and tissue taken deep from cautery.

When the depth of sampling was considered, 81% (39/48) of the muscle samples were negative on both surface and deep analyses. In the remaining samples, 6/48 (13%) were positive only for surface contamination, while 3/48 (6%) were found to be contaminated both superficially and deeply. Of note, five of the nine HEV-positive muscles, including three samples positive on both surface and deep analyses, came from animals negative for the presence of the HEV genome in the liver.

Overall, 4/12 (33%) of the muscle samples taken from animals whose livers had tested positive for HEV were also positive for the virus, while, surprisingly, 5/36 (14%) of the muscle samples from animals with HEV-negative livers showed the presence of HEV RNA.

## 4. Discussion

As reported by the Italian National Institute of Health (Istituto Superiore di Sanità, ISS), 58 cases of hepatitis E were notified in Italy in 2023. Among all cases, 10.8% were diagnosed in Umbria, with the consumption of raw or undercooked pork or WB being the main risk factor [26].

Since the 20th century, the number of WBs has been increasing throughout Europe due to the reintroduction of wild species [27]. Considering the role of these animals as reservoirs of HEV and the related increasing number of infections in humans, systematic surveillance of the wild population of WB is essential.

In the present study, 10.8% of the WB liver samples analyzed tested positive for HEV- RNA. Although this prevalence is significantly lower compared to the 43.6% of the previous hunting season (2021–2022) [16], our results still confirm the high circulation of HEV in WBs in central Italy, in agreement with previous reports about regions bordering Umbria [21,28,29,30]. The absence of long-term studies addressing the epidemiological trend of HEV infection in different WB populations over time and the limited knowledge of the efficacy of the WB immune response against HEV preclude any tentative explanation of the discrepancy observed between the two successive monitoring seasons in the same territory.

However, it opens to some considerations. First of all, there is a need for long-term epidemiological studies in which molecular analysis should be combined with the serological survey to provide information on the circulation of the virus in a given season, together with the seroprevalence in WB populations. It is likely that a high viral load circulating during a specific period, as it may have happened in the 2021–2022 hunting season [16], may promote a protective immune response that limits virus circulation in the following season [31]. Secondly, the majority (66.7%) of the animals analyzed in the present work were older than 23 months and characterized by a positivity rate apparently lower compared to other age groups [16,32,33]. Several studies have reported that seroprevalence increases in adult domestic pigs, whose species is closely related to WB [34,35] and that the percentage of animals positive for HEV in the liver and feces decreases with the age of the pigs [36,37,38,39]. Thus, the age of the WBs should be considered as a risk factor for HEV, suggesting that only older animals should be destined for slaughter for food safety purposes. Finally, the role that climatic variations may play in favoring or not the circulation of certain classes of viruses should not be underestimated [40,41,42]. HEV uses the fecal–oral route to transmit between animals [36], making contaminated water an important vehicle of infection in both WBs and humans. Even if several studies have shown that the prevalence of HEV in the surface water is lower than in wastewater [43,44], it cannot be ruled out that atypical climatic conditions, such as those that characterized the 2022 summer–autumn season in Umbria (https://www.regione.umbria.it/-/servizio-idrografico, accessed on 17 April 2024), may have changed animal habits, particularly in the choice of drinking water sources, increasing the dispersion of WB populations in the territory and reducing the possibility of virus transmission.

WB meat is characterized by its high nutritional quality [45], and its by-products are particularly valued by consumers. Therefore, the demand for WB meat is on the rise and, with it, the risk of HEV infection. HEV infection in WB is considered subclinical and is not identifiable during post-mortem inspection of the animal. Currently, there are no official tests and control policies to identify HEV-positive animals that may, therefore, be overlooked and sent to slaughtering, posing a risk to hunters and consumers.

In a previous study, Feurer and colleagues showed that under natural conditions of infection, pork ham muscles were not positive for HEV [46]. Similarly, none of the samples evaluated by Soares et al. contained HEV genetic material [47]. However, as suggested by the authors, these results could be related to a limited circulation of the virus in the study region or to its presence at undetectable levels in the analyzed products. Conversely, in two different studies led by Di Bartolo and colleagues, a low percentage of pork lingual muscles [48] and WB muscles [17] sampled at the slaughterhouse tested positive for HEV RNA. Thus, although infrequent, the presence of HEV in the muscles of processed carcasses cannot be completely excluded, possibly due to cross-contamination. Cross-contamination may occur by two potential mechanisms, i.e., it may be consequent to the anatomical contiguity between the liver and the different organs manipulated or to the animal’s viremic status. Both situations may provide perfect conditions for HEV cross-diffusion from the liver to muscle, either in the same subject or from infected to uninfected carcasses [49].

In this study, we detected HEV in thigh and loin samples, suggesting that the anatomical localization of specimens may play a major role. In fact, the proximity of the specimen to the liver or gallbladder or the position in which the carcass is placed during exsanguination may promote cross-contamination. Interestingly, however, in our work, positive livers were not always associated with positive muscles and vice versa. In a cross-contamination model proposed by the National Institute for Public Health and the Environment (RIVM) [50], exposure to cutting surfaces during the process of cutting promotes the pathogen transfer from meat to knife and from knife to meat. “The cutting board route”, as named by the authors, represents a critical control point for the management of foodborne hazards in slaughtering. For this reason, good hygiene practices, especially during evisceration and skinning, are essential to avoid HEV dissemination and meat cross-contamination.

**Figure 5:**
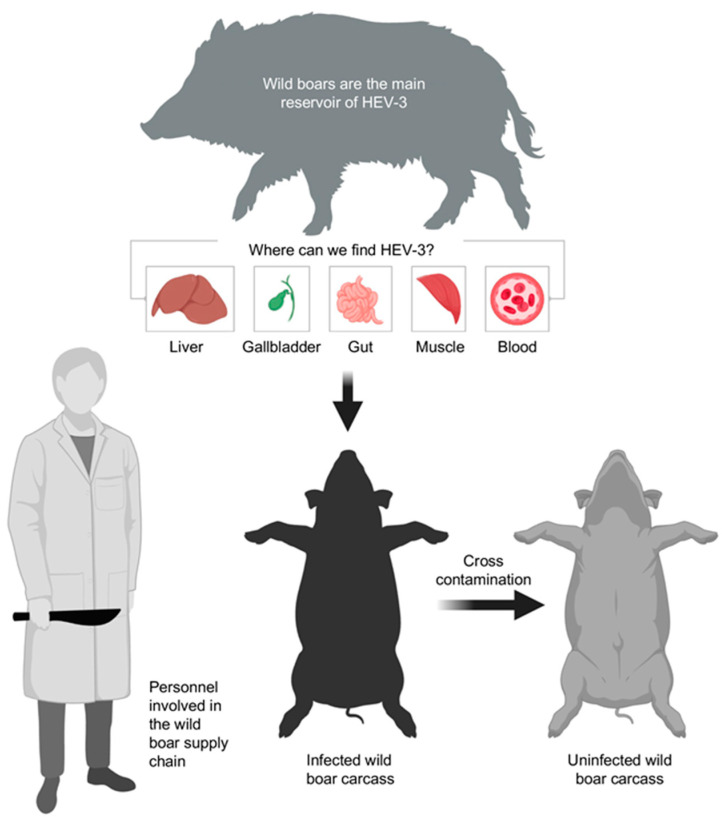
Available data show that the HEV RNA could be detected mostly in the liver and blood but also in fecal and muscle samples [33,48]. Evisceration is recognized within the hazard analysis of the critical control point as a critical control point in hunted game slaughter for management of foodborne hazards due to the high risk of carcass contamination, from liver and blood to meat in the same subject and from infected to uninfected carcasses. The figure was created with BioRender.com.

In this study, 9 of 48 muscle samples were positive for the presence of viral RNA. Among the positive muscle samples, six out of nine were positive for the HEV genome only on the surface, while three out of nine were contaminated, both on the surface and in-depth. It should be noted that five positive muscle samples, including these last three, had been taken from animals whose liver had tested negative. Our results suggest that muscular viral detection—especially on the surface and in muscles of animals whose livers tested negative for HEV (and that were therefore presumably uninfected)—may be caused by improper slaughtering practices that promote cross-contamination (Figure 5).

## 5. Conclusions

In Italy, pork products, whether containing liver or not, have been identified as having a higher risk of HEV transmission [51].

Our work provides results consistent with this assessment. Moreover, the results presented in this study confirm the overall potential risk of HEV cross-contamination on the WB meat slaughter chain in Umbria, central Italy. The risk of having HEV-positive meat is increased by the lack of hygienic measures. Our study emphasizes the need to pay special attention to training hunters and personnel involved in meat processing at the slaughterhouse. The implementation of good hygienic practices at all stages of WB meat processing may help risk managers reduce the impact of HEV and the associated risk to the final consumer.

## Figures and Tables

**Table 1:** Primers and probes used for the detection of HEV.

Target	Primer Name	Primer Sequence	References
ORF3	JVHEVF (forward)	5′-GGTGGTTTCTGGGGTGAC-3′	[22,23]
	JVHEVR (reverse)	5′-AGGGGTTGGTTGGATGAA-3′	
	JVHEVP (probe)	5′-FAM-TGATTCTCAGCCCTTCGC-MGB-3′	

**Table 2:** ATC sample distribution and outcome livers for hepatitis E.

ATC	No. of Positive Livers (%)	% of Livers from the ATC on the Tested Total
ATC1	3/33 (9%)	30%
ATC2	5/53 (9%)	48%
ATC3	4/25 (16%)	22%
Total	12/111 (10.8%)	

**Table 3:** Age distribution and related outcome for liver hepatitis E positivity.

Age Group	No. of Positive Livers (%)	% of Livers Per Age Group
A (0–3 month)	0/2 (0%)	1.8%
B (4–12 month)	1/11 (9%)	9.9%
C (13–22 month)	6/24 (25%)	21.6%
D (>23 month)	5/74 (7%)	66.7%
Total	12/111 (10.8%)	

## Data Availability

The original contributions presented in the study are included in the article, further inquiries can be directed to the corresponding author.
